# Modulation of non-bilayer lipid phases and the structure and functions of thylakoid membranes: effects on the water-soluble enzyme violaxanthin de-epoxidase

**DOI:** 10.1038/s41598-020-68854-x

**Published:** 2020-07-20

**Authors:** Ondřej Dlouhý, Irena Kurasová, Václav Karlický, Uroš Javornik, Primož Šket, Nia Z. Petrova, Sashka B. Krumova, Janez Plavec, Bettina Ughy, Vladimír Špunda, Győző Garab

**Affiliations:** 1 Faculty of Science, University of Ostrava, Ostrava, Czech Republic; 2 Global Change Research Institute, Czech Academy of Sciences, Brno, Czech Republic; 3 Slovenian NMR Center, National Institute of Chemistry, Ljubljana, Slovenia; 4 EN-FIST Center of Excellence, Ljubljana, Slovenia; 5 Institute of Biophysics and Biomedical Engineering, Bulgarian Academy of Sciences, Sofia, Bulgaria; 6 Faculty of Chemistry and Chemical Technology, University of Ljubljana, Ljubljana, Slovenia; 7 Institute of Plant Biology, Biological Research Centre, Szeged, Hungary

**Keywords:** Biochemistry, Biophysics, Plant sciences, Structural biology

## Abstract

The role of non-bilayer lipids and non-lamellar lipid phases in biological membranes is an enigmatic problem of membrane biology. Non-bilayer lipids are present in large amounts in all membranes; in energy-converting membranes they constitute about half of their total lipid content—yet their functional state is a bilayer. In vitro experiments revealed that the functioning of the water-soluble violaxanthin de-epoxidase (VDE) enzyme of plant thylakoids requires the presence of a non-bilayer lipid phase. ^31^P-NMR spectroscopy has provided evidence on lipid polymorphism in functional thylakoid membranes. Here we reveal reversible pH- and temperature-dependent changes of the lipid-phase behaviour, particularly the flexibility of isotropic non-lamellar phases, of isolated spinach thylakoids. These reorganizations are accompanied by changes in the permeability and thermodynamic parameters of the membranes and appear to control the activity of VDE and the photoprotective mechanism of non-photochemical quenching of chlorophyll-a fluorescence. The data demonstrate, for the first time in native membranes, the modulation of the activity of a water-soluble enzyme by a non-bilayer lipid phase.

## Introduction

The primary function of biological membranes is to allow compartmentalization of cells and cellular organelles and, in general, the separation of two aqueous phases with different compositions. The functioning of these membranes, at the basic level, depends on the organization of their lipid molecules into bilayer structures^1–3^. These structures provide a two-dimensional matrix, which is capable of embedding intrinsic proteins and permits the lateral diffusion of mobile compounds inside the 2D matrix of the membrane. By acting as highly selective barrier, the bilayer membrane allows the formation of concentration gradients of ions and other water-soluble compounds across them. The generation and utilization of the transmembrane electrochemical potential gradient for protons, Δµ_H_^+^ or proton-motive force, is of pivotal importance in biological energy conversion^4^.

Most membrane lipids readily form bilayers. However, biological membranes also contain non-bilayer lipid species—which do not self-assemble into bilayers^5^. Their role in the biomembranes is still enigmatic. Most noteworthy, in energy-converting membranes non-bilayer lipids constitute about half of their total lipid content, which is not easy to reconcile with the fundamental role of the bilayer structure in the formation of Δµ_H_^+^ and its utilization for ATP synthesis^6, 7^.

Plant thylakoid membranes, flattened lipid vesicles, accommodate virtually all protein compounds of the light-energy converting apparatus: the two photosystems (PSs), PSII and PSI, along with their associated light-harvesting antenna complexes (LHCII and LHCI, respectively), the cytochrome b_6_f complex and the ATP-synthase. The thylakoid membranes separate the two aqueous phases of chloroplasts, the inner (lumenal) and outer (stromal) sides. The primary charge separation in the photochemical reaction centers and the consecutive vectorial electron and proton transport processes generate a transmembrane ΔpH (acidification of the lumen by 2–3 pH units) and an electric potential gradient (ΔΨ, of approximately 10^5^ V cm^−1^), components of the proton-motive force.

With regard to their lipids, the thylakoid membranes contain monogalactosyl-diacylglycerol (MGDG, ~ 50%), digalactosyl-diacylglycerol (DGDG, ~ 25–30%), sulfoquinovosyl-diacylglycerol (SQDG, ~ 10–15%) and phosphatidylglycerol (PG, ~ 10–15%). However, only DGDG, SQDG and PG, which are cylindrically-shaped lipids, are capable to spontaneously assemble into bilayer (lamellar) structures. In contrast, MGDG, due to its conical shape, preferentially forms non-bilayer phases, such as inverted hexagonal (H_II_), isotropic and cubic phases^6, 8, 9^. Also, lipid mixtures containing more than about 30% MGDG no longer form lipid vesicles^10^.

The ’standard’ fluid mosaic membrane model^2, 3^, with its basic feature of a lipid bilayer embedding membrane proteins, explains all major features of biological membranes. However, this model does not take into account the presence of non-bilayer forming lipids. The lateral pressure model (LPM) complements the standard fluid mosaic model by taking into account that, due to their conical shapes, non-bilayer lipids generate local pressure and maintain a frustrated state in the bilayer membrane, a condition which affects the function of the intrinsic, membrane-integral proteins^11, 12^. Recently, the shortening of chlorophyll-a (Chl-a) fluorescence lifetime in LHCII-containing proteoliposomes was proposed to reflect an increased lateral pressure on LHCII in the adjacent fatty acid region due to the presence of MGDG^13^. By this mechanism, LHCII can be switched from light-harvesting to energy-dissipating regime, a process reminiscent of the in vivo photoprotective mechanism of non-photochemical quenching (NPQ) of Chl-*a* fluorescence^14^. The flexible surface model (FSM), which also challenges the standard model, assigns a key role to non-bilayer lipids in the balance of curvature and hydrophobic forces in lipid–protein interactions and in generating curvature elastic energy, as modulated by the presence of non-bilayer lipids in the bilayer membrane^15^. LPM and FSM both agree that the presence of non-bilayer lipids in the bilayer is important for the functional activity of intrinsic proteins and lends the membranes high plasticity. In these models, however, the formation of non-bilayer phases is restricted to occur only locally and transiently in the membranes, and thus assume no lipid polymorphism of the functional membranes.

An alternative model, the dynamic exchange model (DEM), assumes the co-existence of bilayer and non-bilayer phases and a dynamic equilibrium between different lipid phases^7, 16^. This model is based on two main premises. On the one hand, it has been shown that isolated LHCII and MGDG can form densely packed membranes—showing that intrinsic proteins are capable of forcing non-bilayer lipids into lamellar phase^17, 18^; see also^19^. By this means, the array of protein limits the space available for the bulk lipid phase, impeding the formation of non-bilayer phases inside the membrane. On the other hand, under less favorable conditions, in the absence of such spatial limitation, lipids may segregate out from the bilayer^6, 20–22^. Also, lipid mixtures containing non-bilayer lipids have been shown to assume multiple phases^23^. Molecular dynamics simulations also indicated by the ability of thylakoid lipid mixtures to form stalks, the main intermediate in the transition from a lamellar to H_II_ phase^8^. It is interesting to point out that during stalk formation no clustering of MGDG occurred, suggesting that the phase behaviour of the lipid mixtures is determined by their non-bilayer propensities. According to DEM, non-bilayer lipids and non-lamellar lipid phases help maintaining the homeostasis of membranes, self-regulating its protein-to-lipid ratio via segregation and incorporation of lipids. With the dynamic exchange of lipids between different phases, non-lamella forming lipids and non-bilayer lipid phases have been proposed to contribute to the structural flexibility of membranes^7, 16^.

By using ^31^P-NMR fingerprinting of the phase behaviour of phosopholipids^24^, it has been shown that non-bilayer phases can be induced in lyophilized and reconstituted plant thylakoid membranes upon removing their oxygen-evolving complex or at elevated temperatures^25, 26^. In this case, in thylakoids, the measurements provided information on the phase behaviour of PG molecules in the bulk. (Although a minor component of the thylakoid membrane, only about 10% of the lipid content in a highly protein-rich membrane, PG has been shown to report on the characteristic phase behaviour of the bulk lipid mixture.) ^31^P-NMR spectroscopy experiments on intact thylakoids provided clear experimental evidence for the co-existence of the bilayer and an isotropic, non bilayer lipid phase in isolated, fully functional membranes^27^. The polymorphism of lipid phases was substantiated by polyphasicity of the fluorescence lifetime of the lipophilic probe merocyanine 540 (MC540) incorporated in the thylakoid membranes^28^. Our recent ^31^P-NMR experiments have shown the presence of a bilayer phase and three non-bilayer phases—two isotropic and an inverted hexagonal phase (H_II_). Heterogeneity of lipid phases has also been confirmed by time-resolved spectroscopy of MC540 fluorescence^29^. The different lipid phases have been shown to exhibit large variations upon changing the temperature^27^ and the physico-chemical environment (pH, osmotic and ionic strengths) of the membranes^29, 30^. The gradually increased intensity of the isotropic phases has been correlated with a gradual loss of the impermeability of the membrane, suggesting that the rate of the basal ion flux across the membrane is regulated by non-bilayer lipid phases^31^.

Presently only tentative assignments can be given concerning the origin of the different lipid phases in thylakoids. The H_II_ phase has been proposed to originate from expelled ’free’ lipids, which assemble into structures loosely associated with the membranes. One of the two isotropic phases has been hypothesized to be associated with the fusion of membranes, such as, in vascular plants, at the junction of the granum and stroma membranes^32, 33^. Isotropic signal is also proposed to originate from molecular assemblies formed between lipids and water-soluble lipocalin or lipocalin-like proteins, such as the key enzymes of the xanthophyll cycle, violaxanthin de-epoxidase (VDE) and zeaxanthin epoxidase^34^, and the plastid lipocalin LCNP^35, 36^. VDE and LCPN are located in the thylakoid lumen and participate in NPQ of Chl-*a* fluorescence, whereas zeaxanthin epoxidase (ZE) is found on the stroma side^37^. Indeed, in vitro experiments have revealed that VDE at low pH binds MGDG^38^. Moreover, by using model membranes and different mixtures of bilayer and non-bilayer lipid species, it has been shown that the activity of VDE, i.e. the de-epoxidation of violaxanthin (Vx) to anteraxanthin (Ax) and zeaxanthin (Zx) depends strictly on the presence of non-bilayer lipid phase, proposed to be a H_II_ phase^39, 40^. However, evidence for the correlation between the non-bilayer lipid phases and the activity of VDE in the native thylakoid membranes is still missing. Our understanding is even less advanced as concerns the role of non-bilayer lipid phases in the structural and functional plasticity of the thylakoid membranes under physiologically relevant conditions.

In the present study, by using ^31^P-NMR spectroscopy and short acquisition times on freshly isolated spinach thylakoid membranes, we were able to induce temperature- and pH-dependent changes in the lipid-phases that remained largely reversible. Complementary experiments under comparable conditions revealed that these changes in the lipid-phase behaviour of thylakoids were associated with pronounced reversible changes in the permeability of the membranes. Variations in the lipid phases brought about reversible enhancements in the de-epoxidation state of xanthophylls—providing evidence for the regulation of the activity of VDE in its native thylakoid membrane by non-bilayer, isotropic lipid phases, which also modulated the magnitude of NPQ.

## Results and discussion

### ^31^P NMR fingerprints of lipid polymorphism in thylakoid membranes

**Figure 1:**
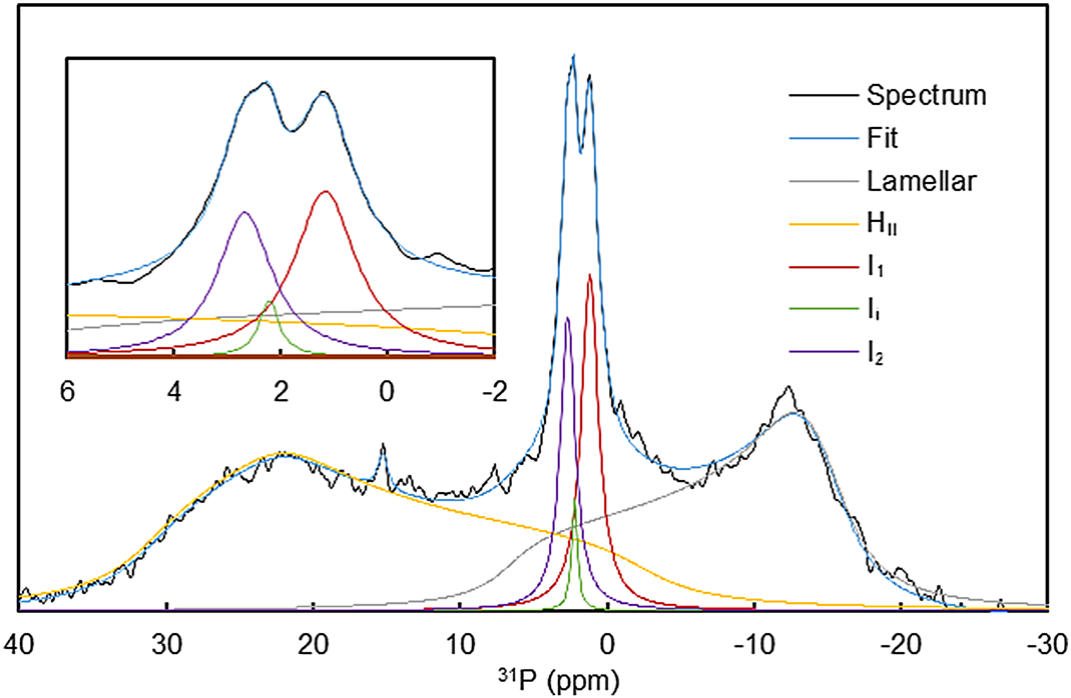
Measured and spectrally deconvoluted and fitted ^31^P-NMR spectra of isolated spinach thylakoid membranes. Contributions from different lipid phases were determined via deconvolution of the measured spectra (black curves) into spectral components characteristic of the lamellar, inverted hexagonal (H_II_) and isotropic phases (I_1_, I_2_ and I_i_)—as indicated, using the software DMfit. The fitted spectra are also plotted (blue lines); inset shows the spectrum in the isotropic region. Chl concentration, 6.7 mg/ml; number of scans, 7,500.

The capability of ^31^P-NMR spectroscopy to identify different lipid phases in artificial and native systems has been thoroughly documented (e.g.^41, 42^). This technique has revealed the polymorphic phase behaviour of isolated plant thylakoid membranes and uncovered that non-lamellar lipid phases play an active role in the structural dynamics of thylakoid membranes^27, 29^. As reported earlier^29^ and shown in Fig. 1, the ^31^P-NMR spectra of isolated spinach thylakoid membranes typically contain four well discernible phases: a lamellar phase (L) with a broad band peaking at around − 10 ppm, two isotropic phases (I_1_ and I_2_), displaying relatively sharp bands, typically around 2 and 4 ppm, respectively, and an inverted hexagonal phase (H_II_), giving rise to a broad band peaking at ~ 25–30 ppm. Deconvolution of the spectrum, using the spectral components for L, I and H_II_ phases, reveals a third, relatively weak isotropic peak between I_1_ and I_2_; this intermediate peak hereafter will be referred to as I_i_. Albeit the spectroscopic fingerprints varied from batch to batch, virtually all spinach thylakoid preparations (n > 50), isolated from leaves purchased on different local markets and in different seasons, contained all the four phases (or sometimes well discernibly also the fifth (I_i_) phase).

### pH-dependent changes in the lipid polymorphism and in the membrane organization

**Figure 2:**
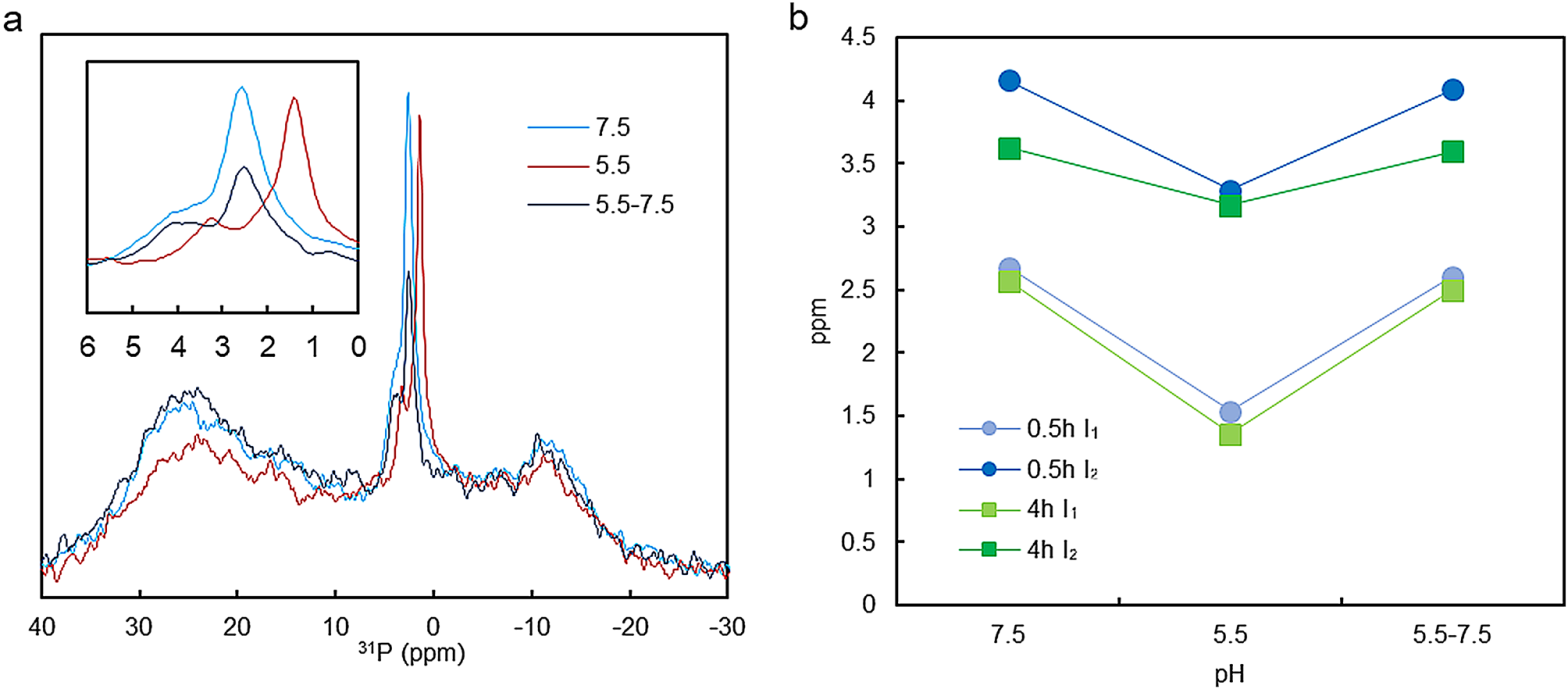
Effects of low-pH treatment on the ^31^P-NMR spectra of isolated spinach thylakoid membranes and the peak positions of I_1_ and I_2_. (**a**) Spectra measured on thylakoid membranes in sorbitol-based media at pH 7.5 and 5.5, and 7.5 after a 5 min incubation time at pH 5.5 (5.5–7.5)—as indicated; see Methods. Spectra are normalized to equal Chl concentrations and to same number of scans; for individual Chl concentrations see Figure S1. Inset, the isotropic region. (**b**) Peak positions of the two isotropic peaks, obtained from mathematical deconvolution, as a function of pH treatment; the peak positions plotted were determined 30 min and 4 h after the start of the measurement. The individual spectra, together with the mathematically deconvoluted components are shown in Figure S1; the series of spectra of samples at pH 5.5, recorded during storage of the sample in the dark at 5 °C, are displayed in Figure S2.

As reported earlier, exposing the thylakoid membranes to low pH induces significant changes in the lipid-phase behaviour of membranes, affecting primarily the isotropic phases^29^. Here we show that the changes are largely reversible (Fig. 2a). Deconvolution of the spectra, resolved all three isotropic bands as well as the bands arising from the L and H_II_ phases (Supplementary Fig. 1a–c). This analysis revealed that the isotropic peak positions shifted to higher field values upon lowering the pH from 7.5 to 5.5; these shifts were almost fully reversible when the pH was raised again to 7.5 (Fig. 2b). Smaller but otherwise similar reversible shifts were obtained with pH 6.5 (data not shown). These changes cannot be accounted for by a pH dependence of the chemical shifts of PG molecules but evidently originate from changes in the physico-chemical environments of the lipid molecules in the bulk: (1) the peak positions, both at neutral and acidic pH, were shifted gradually in the same direction during storage in the dark (Fig. 2b and Supplementary Fig. 2; see also^31^); (2) the magnitude of the pH-induced shifts and the stabilities at pH 7.5 and pH 5.5 depended significantly on the composition of the suspending medium (NaCl vs. sorbitol as osmoticum) (Supplementary Fig. 2). Also, literature data on the pH-dependence of the ^31^P-NMR signatures of phospholipid micelles revealed pH dependent chemical shifts only in the alkalic and highly acidic regions^43, 44^; between pH 5.5 and 7.5, instead, phase transitions were observed^45^.

It is also interesting to note that parallel to the dramatic variations in the isotropic phases, no low-pH induced enhancement could be seen in the H_II_ phase. As can be seen in Supplementary Fig. 1, and other unreported experiments, the overall intensity of the H_II_ phase relative to the L phase did not increase. (This observation will be used in the section related to VDE activity.)

**Figure 3:**
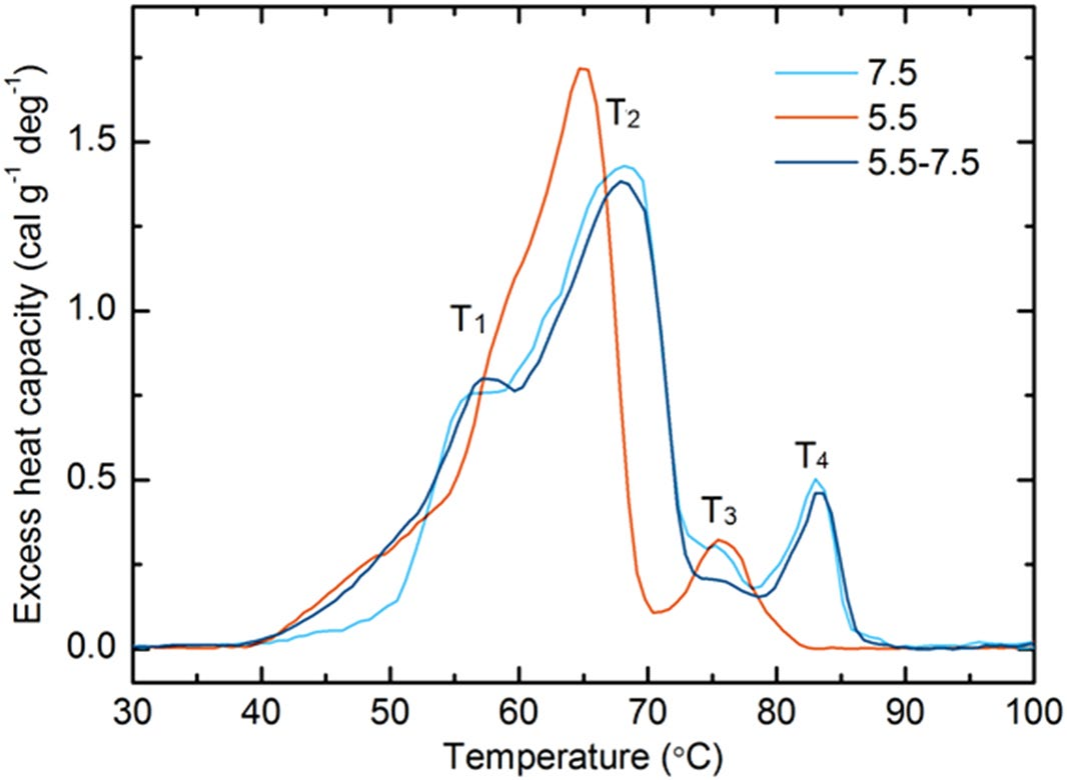
Effect of pH treatments on the DSC thermograms of isolated spinach thylakoid membranes. Samples with Chl concentration 1.3 mg/ml were linearly heated with a rate of 0.5 ºC/min. DSC scans are shown for samples incubated at pH 7.5 (cyan), 5.5 (red) and 7.5 after pH 5.5 (blue) treatment. Incubation time at pH 5.5 was 40 min. For clarity the well resolved calorimetric transitions for the sample at pH 7.5 are denoted as T_1_–T_4_.

The changes in the lipid phases occurring upon the transition from pH 7.5 to pH 5.5 were correlated with pronounced and largely reversible destabilization (i.e. downshift) of the main calorimetric transition (from 67 to 65 °C) in spinach thylakoid membranes (Fig. 3 and Supplementary Table 1); this transition can be assigned to LHCII denaturation based on our previous works^46–48^. The low-pH induced shift in the last thermal transition in the thylakoids’ DSC profiles was even more pronounced (from 83 to 78 °C), but will not be commented further since the origin of this thermal event is yet unclear. Calorimetry is routinely used to monitor lipid phase transitions^49^. However, in the highly organized system of plant thylakoid membranes, changes in the DSC band-structure are by far dominated by proteins and their macro-arrays^46^. Nevertheless, their light- and thermal stabilities are strongly affected by the lipid environment of the protein complexes^18, 50–53^. In other terms, the thermodynamic features of photosynthetic complexes are strongly affected by the chemical and physical properties of the lipid matrix that surrounds them. Regarding the role of non-bilayer lipids, correlation between the lipid composition of thylakoid membranes and their thermal stabilities has been shown by using steady-state and time-resolved spectroscopy measurements on MGDG-enriched (*Arabidopsis dgd1* mutant) thylakoid membranes, which revealed that the extrusion of lipids at elevated temperatures was accompanied by an increased membrane permeability and a higher susceptibility of thermal destabilization of the protein complexes^47^. Vice versa, co-solute (2 M sucrose) induced extrusion of lipids from the membrane, which produced H_II_ phases^20, 29^, led to an improved thermal stability of PSII and the protein macrodomains^30^.

It is interesting to note that lowering the pH led to a significant increase in the membrane permeability, which was manifested in accelerating the relaxation of ΔΨ via basal ion flux across the membrane^54^, as reflected by the electrochromic absorbance transients at 515 nm (Table 1). These changes also showed considerable degree of reversibility when the previously low-pH treated samples were returned to neutral pH (Table 1, Supplementary Fig. 3). In our earlier study we have shown close correlation between the gradually enhanced isotropic lipid phases, accompanied by shifts of the ^31^P-NMR isotropic peaks to higher field positions, and the increased permeability of thylakoid membranes^31^.

**Table 1:** Relative half-decay times (t_1/2_) of the ΔA_515_ electrochromic absorbance changes of isolated spinach thylakoid membranes after different pH treatments.

pH	Relative half-decay time (± SD)
7.5	100^a^
5.5	9 (± 4)^b^
7.5–7.5	92 (± 12)^a^
5.5–7.5	42 (± 23)^c^

Mean values and standard errors from three independent experiments. Different letters indicate statistically significant differences (ANOVA, F-test, *P* < 0.05). For typical kinetic traces see Supplementary Fig. 3. t_1/2_ at pH 7.5 was 0.41 ± 0.25 s (n = 3). Chl concentration, 20 µg/ml.

### De-epoxidation of the xanthophyll pigments

Since VDE is activated at low pH and its functioning depends on the presence of non-bilayer lipid phases, it was of special interest to test the stepwise conversion of Vx to Ax and Zx and its correlation with changes in the lipid phase behaviour of thylakoid membranes. Shown in Table 2, are variations in the amounts of Vx, Ax and Zx in the dark and upon 10 min illumination, as well as changes in the de-epoxidation state (DEPS = (Ax + Zx)/(Vx + Ax + Zx) of the xanthophyll cycle pigments. As expected, in the dark at pH 5.5, a significant degree (34 ± 7%) of de-epoxidation occurred, but not at pH 6.5 (data not shown) and higher. Upon illumination of samples, moderate DEPS values (24 ± 9%) were obtained at pH 7.5, whereas at pH 5.5 DEPS in illuminated samples (37 ± 6%) remained almost the same as in dark adapted thylakoids. All de-epoxidation reactions were fully blocked in the presence of 5 mM dithiothreitol (DTT), inhibitor of VDE activity^55^ (data not shown).

**Table 2:** Effect of different pH treatments on the composition of the xanthophyll cycle pigments in isolated spinach thylakoid membranes and the de-epoxidation states (DEPS) in the dark and after illumination at 5 °C.

	Vx (± SD)	Ax (± SD)	Zx (± SD)	DEPS (± SD)
**Dark**				
7.5	100^a^	0^a^	0^a^	0^a^
5.5	66 (± 7)^b^	17 (± 3)^b^	17 (± 10)^b^	34 (± 7)b
7.5–7.5	100^a^	0^a^	0^a^	0^a^
5.5–7.5	100^a^	0^a^	0^a^	0^a^
**Light**				
7.5	76 (± 9)^ab^	16 (± 2)^a^	8 (± 7)^ab^	24 (± 9)^ab^
5.5	63 (± 6)^a^	17 (± 2)^a^	20 (± 8)^a^	37 (± 6)^b^
7.5–7.5	83 (± 8)^b^	14 (± 4)^a^	2 (± 5)^b^	17 (± 8)^a^
5.5–7.5	85 (± 8)^b^	13 (± 5)^a^	2 (± 4)^b^	15 (± 8)^a^

Xantophyll pigment contents (% of total xanthophyll cycle pigments) and DEPS (= (Ax + Zx)/(Vx + Ax + Zx), %) in dark-adapted thylakoids (Dark) and after 10 min of 770 μmol photons m^−2^ s^−1^ illumination (Light). Mean values and standard errors from 3 to 7 independent experiments. Different letters in columns indicate statistically significant differences (ANOVA, F-test, *P* < 0.05).

With regard to the role of non-bilayer lipid phases, we stress that the requirement of a non-bilayer lipid phase is satisfied already at neutral pH. Also, as pointed out above, the most characteristic low-pH induced variations occur in the isotropic phases, rather than in the H_II_ phase. This suggests that the non-bilayer lipid phase required for the activity of VDE is an isotropic phase, in accordance with the tentative assignment of one of the isotropic phases being a VDE:lipid assembly^29^. This is at variance with the data in model systems, where the non-bilayer phase was identified as H_II_ phase^39^. It has been proposed that VDE, as a lipocalin-like water soluble enzyme, is capable of binding substantial amounts of lipids, which thus may form a (an isotropic) shell around this protein^29^.

Because the experiments were performed at 5 °C, where the activity of VDE is low, illumination of the thylakoids membranes at pH 7.5 induced only slight de-epoxidation, particularly Ax was formed (Table 2). In contrast, at pH 5.5 considerably higher amounts of Zx were formed in the dark and illumination did not enhance sizeably the DEPS. These data agree with those of Goss and coworkers^56^, who reported that pH 5 is sufficient to induce the maximum DEPS (including Zx formation). It is also noteworthy that the de-epoxidation in darkness was fully reversible when the pH of thylakoid suspension was adjusted back to 7.5, whereas no such rapid epoxidation of xanthophyll pigments was observed after re-darkening of illuminated thylakoids independently of the pH (data not shown). These data strongly suggest that the accumulation of Ax and Zx at low pH in the dark is directly associated with the modulation of the isotropic lipid phases. Also to be noted, the light-induced de-epoxidation of thylakoid membranes exposed to the sequence of pH 7.5 to 5.5 and back to 7.5 was essentially the same as in thylakoids exposed only to pH 7.5—demonstrating that the low-pH induced changes were fully reversible. The proposed correlation between changes in the isotropic lipid-phases and the activity of VDE will be further supported by data on thermally induced membrane reorganizations and the enhancement of VDE activity.

### Temperature-dependent changes in the lipid polymorphism and in the membrane organization

The role of lipid molecules and lipid phase transitions, including non-bilayer lipid phases, in thermal effects on the structure and function of biological and model membranes has for long been in the focus of research^1, 57–59^. Also in oxygenic photosynthetic organisms, temperature is one of the most important environmental factors. Growth temperature controls the lipid composition and, vice versa, the lipid composition of thylakoid membranes largely determines the physiological range and temperature-stress tolerance of the organisms^60–62^.

Earlier temperature-dependence experiments of lipid phases of isolated thylakoid membranes, using the technique of ^31^P-NMR spectroscopy, revealed that elevating the temperature in the physiological range (< 30 °C) led to a gradual loss of the L phase, accompanied by a stepwise increase of the isotropic signals^27^. Capitalizing on the much better S/N ratio of modern spectrometers, allowing to reduce the data acquisition time from 1 h to 10–15 min, at the same time, requiring much less membranes, we reinvestigated this question. Data in Supplementary Fig. 4, while allowing the identification of all lipid phases even at 25 °C, confirm the weakening of the L and H_II_ phases and the enhanced intensities in the isotropic region upon gradually increasing the temperature from 5 to 15 and to 25 °C.

**Figure 4:**
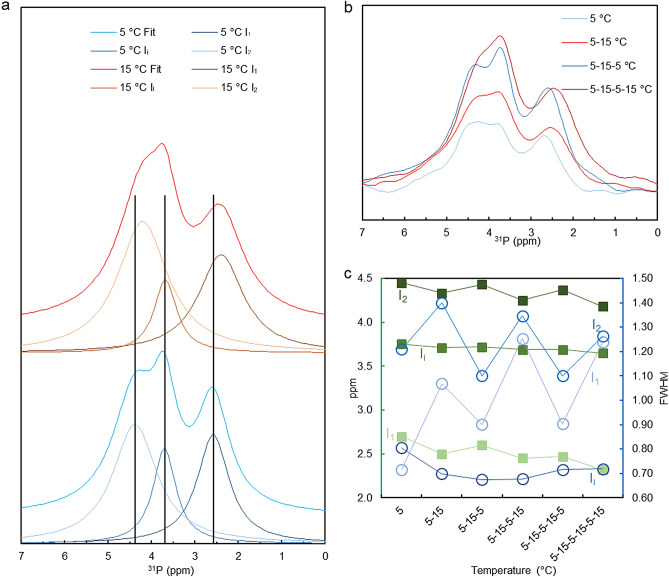
^31^P-NMR spectral characterization of temperature-dependent variations of the isotropic lipid phases of isolated spinach thylakoid membranes. (**a**) Spectra obtained after averaging 3 cycles of switching the temperature between 5 and 15 °C; also shown, the deconvoluted component spectra. The vertical lines mark the peak positions at 5 °C. (**b**) Spectra recorded during the first two cycles of switching the temperature between 5 and 15 °C. The Chl concentration was 7.8 mg/ml and the number of scans was 1,600 for each spectrum. (**c**) Variations of the peak positions (full squares) and bandwidths (open circles) at half maxima (FWHM) during the thermal cycle.

More importantly, the better S/N ratio and faster isolation and data acquisition times, allowed us to test the reversibility of the temperature-dependent variations of the lipid phases. Figure 4a displays the averaged spectra from a series of experiments in which the temperature was cyclically switched between 5 and 15 °C. This figure shows that upon changing the temperature from 5 to 15 °C, the isotropic peaks I_1_ and I_2_ are shifted upfield. This could be recognized despite the time-dependent drifts of the peaks in the same direction (cf.^31^). Reversibility of the changes induced by increasing the temperature from 5 to 15 °C can be clearly recognized in the isotropic region (Fig. 4b), despite the drifts in the intensities and peak positions, which also occur at constant temperatures. Indeed, the peak positions of the I_1_ and I_2_ isotropic bands alternated between their low- and high-field positions, at 5 and 15 °C, respectively (Fig. 4c). At the same time, the peaks at 15 °C were considerably broader, a fact that could be explained by the expected increase in the dynamics of phospholipid transition between different phases at higher temperature. (Deconvolution of the spectra for the first 5 and 15 °C spectra are shown in Supplementary Fig. 5).

The temperature–induced variations in the isotropic lipid phases were associated with dramatic changes in key parameters of the thylakoid membranes; most particularly, in the permeability of membranes. The shortening of the half-decay time of ΔA_515_ (Supplementary Table 2) followed a similar pattern as the changes in the isotropic lipid phases. In general, the acceleration of the decay of the membrane potential at higher temperatures can be correlated with the higher mobility of the lipid molecules in the bilayer; this has been reported also on thylakoid membranes^63^. Although such an effect might be explained without invoking changes in the lipid-phase behaviour of thylakoid membrane, in broad terms, these data are also in harmony with our earlier hypothesis^31^ concerning the correlation between the enhancement and shift of the isotropic phases and the relaxation of ΔΨ.

**Figure 5:**
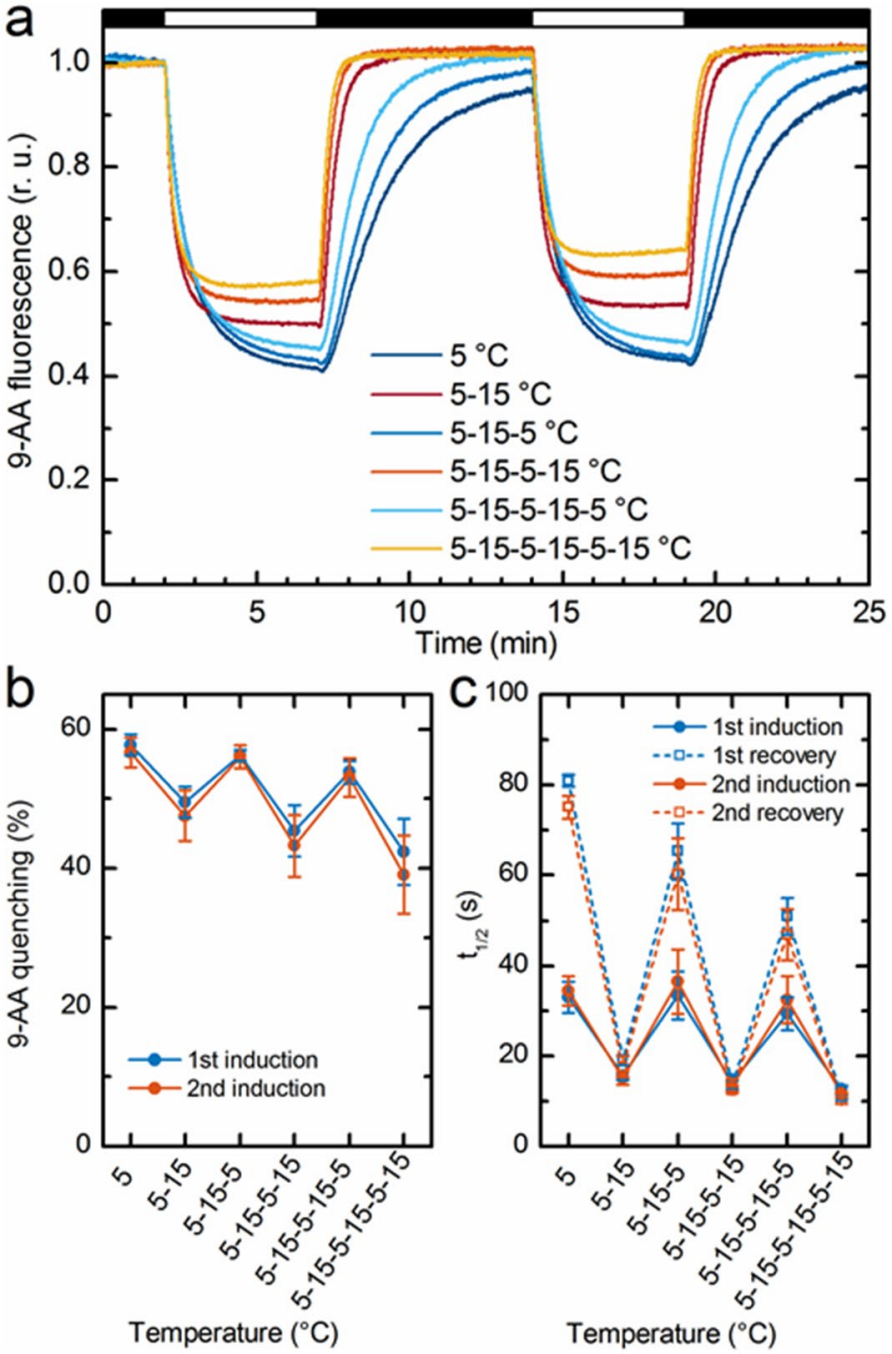
Variations of the magnitude and kinetic parameters of the light-induced pH gradient across isolated spinach thylakoid membranes during 3 cycles switching the temperature between 5 and 15 °C; ΔpH was measured with the aid of fluorescence quenching of 9-AA. (**a**) Typical kinetic traces recorded during two dark–light cycles, as indicated by black and white horizontal bars, respectively. (**b**) The depth of quenching during the first and second illumination periods with 5 min of light of 770 μmol photons m^−2^ s^−1^ followed by 7 min of dark relaxation. Mean values ± SD, obtained from 3 independent experiments. (**c**) Rise and decay halftimes from the same series of experiments. Chl concentration, 25 µg/ml.

The conclusion on the increased permeability of the membranes at 15 °C relative to that at 5 °C is further supported by the analysis of 9-AA (9-amino acridine) fluorescence quenching kinetics, reflecting the light-induced generation and relaxation of ΔpH^64^. Indeed, as shown in Fig. 5a, the relaxation of 9-AA quenching is accelerated upon raising the temperature from 5 to 15 °C. As illustrated in Fig. 5b, the depth of quenching—with some drifts—alternated between the two states, following the cyclical changes in the temperature between 5 and 15 °C. The same held true for the rise and decay half-times, which were faster at 15 °C compared to 5 °C (Fig. 5c).

### Temperature-dependence of the light-induced de-epoxidation of the xanthophyll pigments

**Figure 6:**
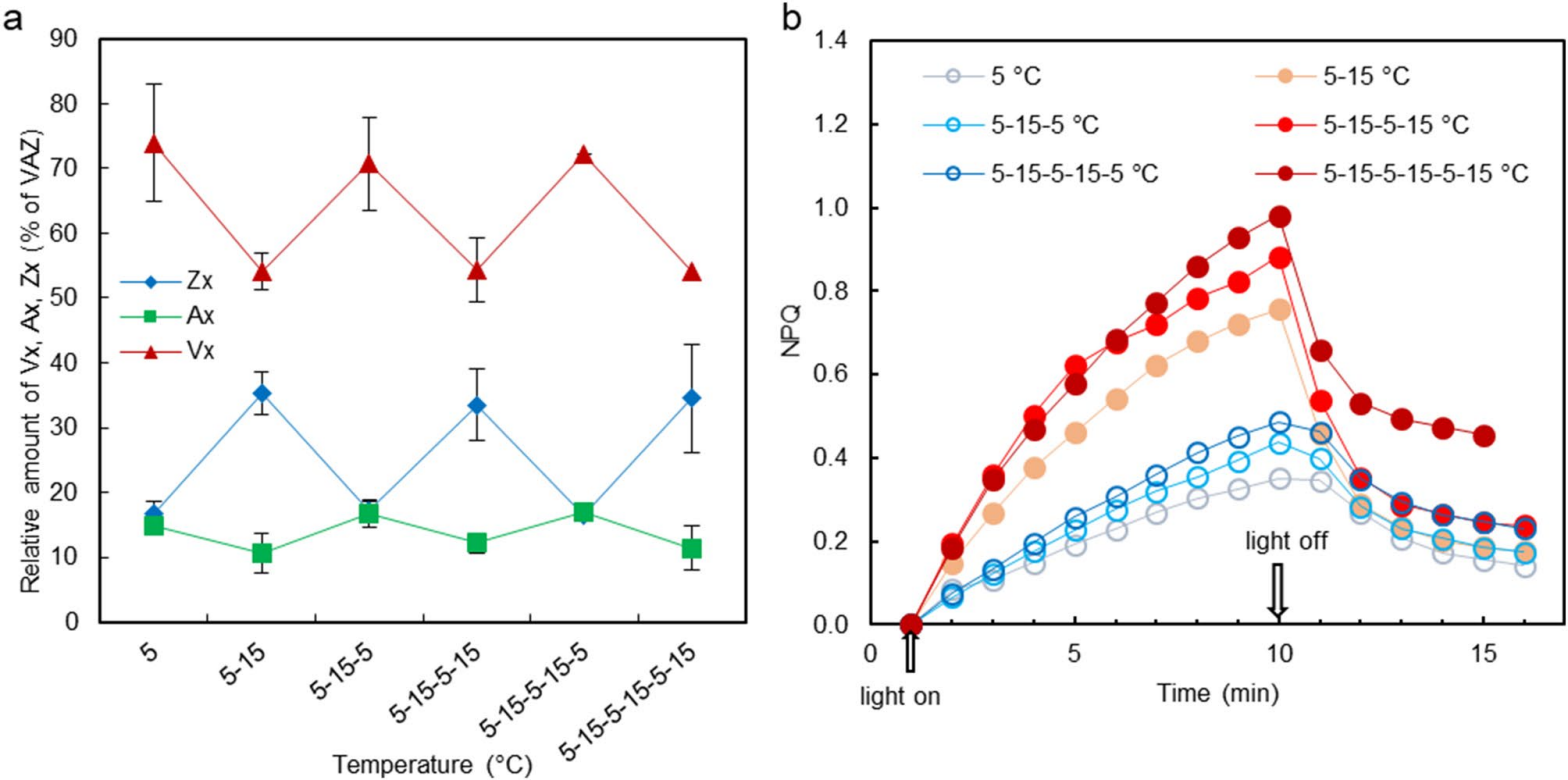
Temperature-dependent variations of the light-induced de-epoxidation of violaxanthin and the NPQ of isolated spinach thylakoid membranes upon cyclically switching the temperature between 5 and 15 °C. (**a**) Relative amounts of the xanthophyll-cycle pigments Vx, Ax and Zx at the end of the illumination periods (770 μmol photons m^−2^ s^−1^ for 10 min). Mean values and standard errors (n = 3). (**b**) Typical rise and decay kinetics of NPQ under the same illumination conditions. Chl concentration, 25 µg/ml.

The thermally induced reversible enhancement of the ^31^P-NMR isotropic resonances and the peak shifts to higher-field positions were associated with enhanced de-epoxidation rates of the thylakoid membranes—as shown by the cyclic variation of the xanthophyll-cycle pigments upon cyclically changing the temperature between 5 and 15 °C (Fig. 6a). Strong temperature dependence of VDE activity has earlier been reported for intact leaves, with very high, 105 kJ/mol, activation energy values at 15 °C compared to 57 kJ/mol at 30 °C^65^. This again suggests the involvement of lipid phase transition: the enhancement of non-bilayer lipid phases at elevated temperature.

The rise kinetics, magnitude and relaxation of NPQ are also strongly modulated by the temperature (Fig. 6b)—similar to earlier data in pea leaves^66^. Interestingly, in thylakoid membranes DTT sensitivity of NPQ could be discerned only at 15 °C and despite the sizeable accumulation of Zx during illumination, no Zx-dependent NPQ could be detected at 5 °C (Supplementary Figure 6). This, again, might be explained due to the trapped conformational state of the membranes at low temperature^66^. In general, these NPQ data are in harmony with the allosteric regulation of fast, energy-dependent component, qE of NPQ^67^, and emphasize the importance of structural plasticity of thylakoid membranes, which—as shown here and in our previous works—is largely governed by the presence of non-bilayer lipids and the polymorphic phase behaviour of the thylakoid membranes.

In order to justify the central role of non-bilayer lipid phases in VDE activity, we must assess the effects of the other factors governing its activity; namely, the formation and preservation of ΔpH. First, we point out that the magnitude of the light-induced ΔpH is very similar at 5 and 15 °C, and although the rate of its generation is slower at 5 °C, the same level is reached with very little difference between 5 and 15° C. (In fact, the 9-AA quenching is more pronounced at 5 °C, but that is evidently due to its slower relaxation—Fig. 5). The faster relaxation rate of the proton gradient at 15 °C, relative to 5 °C, should, in principle, decrease the efficiency of de-epoxidation of Vx. The fact that DEPS is significantly higher at 15 °C than at 5 °C, shows that this factor, i.e. the substantially increased permeability of the membranes, is not only compensated but is overruled by the other key factor, the non-bilayer lipid phase. Hence, it appears that the specific changes in the non-bilayer lipid phases determine the temperature-dependence of the activity of VDE.

## Conclusions and perspectives

In this study, we revealed characteristic low-pH and thermally-induced reversible enhancements and peak shifts of the isotropic phases in isolated spinach thylakoid membranes. These variations were associated with reversible modifications in the membrane structure, most notably increasing their permeability upon lowering the pH and increasing the temperature. The low-pH and thermally induced changes in the isotropic lipid phases showed close correlation with the activity of VDE in the thylakoid membranes. To our knowledge, this is the first demonstration of a non-bilayer lipid phase, in the native membrane, regulating the activity of a water-soluble enzyme—a finding which is in reasonable agreement with earlier data obtained in artificial membranes.

In general, as shown here and in our earlier works, non-bilayer lipid phases significantly contribute to the structural dynamics of fully functional thylakoid membranes, which, in turn, appears to fine-tune the functional activity of membranes. Thylakoid membranes, in this regard, are not unique—recent experiments have revealed the emergence of non-bilayer isotropic phases in bovine-heart inner mitochondrial membranes parallel with their ability to synthesize ATP^68^. Hence, these data demonstrate the physiological importance of non-bilayer lipids and non-lamellar lipid phases both in animal mitochondrial and plant thylakoid membranes, two representatives of energy-converting biological membranes.

## Materials and methods

### Isolation of thylakoid membranes

Spinach thylakoid membranes were isolated as described earlier^29^. Briefly, spinach leaves, purchased on the local market and stored for 1–3 days at 4 °C in the dark before use, were homogenized in buffer A (50 mM Tricine pH 7.5, 5 mM MgCl_2_, 5 mM KCl, 400 mM sorbitol). The suspension was filtered through four layers of cheese cloth and centrifuged for 2 min at 400*g*. Next, the supernatant was centrifuged for 10 min at 6,000*g*. The chloroplasts were osmotically shocked in a hypotonic buffer B containing 50 mM Tricine (pH 7.5), 5 mM MgCl_2_ and 5 mM KCl for 10 s, followed by the immediate addition of buffer 2A containing double-strength osmoticum (800 mM sorbitol and 50 mM Tricine pH 7.5, 5 mM MgCl_2_ and 5 mM KCl) before centrifugation for 10 min at 6,500*g*. The pellet was finally resuspended in buffer A of appropriate pH. In case of pH reversibility measurements, after resuspension in reaction medium with changed pH and 5 min incubation time, the suspension was centrifuged for 10 min at 6,500*g* and resuspended in buffer A (pH 7.5). In some experiments, instead of the sorbitol-based reaction media, we used a NaCl-based medium, as described earlier^31^. (For DSC measurements, a somewhat different protocol was used—see below.)

For the measurements of temperature-induced changes, the final suspension of thylakoids in buffer A (pH 7.5) was placed into heating/cooling dry block (CH-100, Biosan, Riga, Latvia) and aliquots were taken for dilution in buffer A of respective temperature for all techniques except NMR spectroscopy where the temperature was controlled directly inside the sample holder of the spectrometer. The temperature was switched between 5 and 15 °C in three cycles. The Chl content of the samples was determined according^69^. All these procedures were performed on ice under dim light.

### ^31^P-NMR measurements

^31^P-NMR measurements were performed as described earlier^29^. In detail, spectra were recorded on DD2 600 MHz NMR spectrometer (Agilent) using a OneNMR probe and Avance Neo 600 MHz NMR spectrometer (Bruker) using a BBFO probe, both tuned at the resonance frequency of the ^31^P nucleus, with 5 mm outer diameter tubes containing about 1.2 ml thylakoid suspension at a Chl content of about 8–10 mg/ml. As tested earlier, at this concentration, no magnetic orientation of the membranes occurs^27^. The temperature was controlled within 0.1 °C; spectra were recorded using a 40°rf pulse, an interpulse time of 0.5 s and no ^1^H-decoupling. ^31^P chemical shifts are reported relative to 85% H_3_PO_4_ in water (*δ*P = 0 ppm) used as an external reference. Spectral deconvolutions were performed by using the DMfit software^70^.

### Electrochromic absorbance changes

Electrochromic absorbance changes (ΔA_515_), induced by single-turnover saturating (0.5 J) Xe flashes of 2 μs duration at half-peak intensity (Hamamatsu LF1 L-11730-04-01-1, Shimokanzo, Japan) were recorded at 520 nm and 546 nm using Joliot-type kinetic spectrometer JTS-100 (Biologic, France). Kinetic traces were measured on dark-adapted isolated membranes at a Chl content of 20 µg/ml in a cell with optical pathlength of 1 cm. In order to improve the signal-to-noise ratio, 5 kinetic traces were collected with a repetition rate of 0.15 s^-1^ and averaged; it was checked that repetitive flashes did not exert any effect on the ΔA_515_ kinetics. The measurements were repeated on three independently isolated thylakoid membranes.

### NPQ measurements

NPQ was determined from Chl-*a* fluorescence measurement using PAM 101 fluorometer (Walz, Effeltrich, Germany) equipped with a DW2/2 electrode chamber for measurement on liquid samples (Hansatech Instruments, United Kingdom). Isolated thylakoid membranes were diluted to a Chl content of 25 µg/ml by buffer A of the appropriate pH. Methyl viologen (50 μM) as artificial electron acceptor and ascorbate (30 mM) as co-substrate of VDE were also added. Chl-*a* fluorescence was recorded during the 15 min period of the different pH conditions (see above) and at temperatures of 5 and 15 °C (as in the ^31^P-NMR measurements). NPQ was induced by actinic illumination (10 min; 770 µmol photons m^−2^ s^-1^); the relaxation of NPQ was monitored during a 5 min dark period after the termination of illumination. Saturating light flashes (3,900 μmol photons m^−2^ s^−1^) with a duration of 800 ms were applied every minute. NPQ was calculated as Stern–Volmer quenching (*Fm/Fm′* − 1) according to^71^.

### Fluorescence quenching of 9-AA

To measure the 9-AA fluorescence, Xe-PAM fluorometer (Walz, Effeltrich, Germany) and a cold light source (KL1500 Schott) for white light actinic illumination (770 μmol photons m^−2^ s^−1^) were used. An interference filter (XHQA365, Asahi Spectra, Tokyo, Japan) and an emission filter BG39 (Schott, Jena, Germany) together with UV absorbing filter (Lee 226 UV filter, Lee Filters, UK) were used in the excitation and emission pathways, respectively. By this means, the sample was excited by a weak measuring light in the wavelength range of 360–370 nm and fluorescence emission was detected between 400 and 640 nm. Reactions were performed in quartz glass cuvette with optical pathlength of 1 cm and with a 2 ml reaction volume containing 50 mM Tricine (pH 7.5), 5 mM MgCl_2_, 5 mM KCl, 400 mM sorbitol, 20 µM methyl viologen, 10 µM 9-AA, and thylakoids at 20 µg Chl/ml. The measurement frequency of the XE-PAM was 64 Hz, with a gain of 12 and a damping of 1 (device parameters). Temperature control was performed using a Peltier thermostat F-3004 (Horiba Jobin Yvon, Paris, France). The time course of 9-AA fluorescence quenching and recovery were fitted using kinetic equations (single exponentials). This analysis yielded the maximal quenched level (Fq), the half-time of fluorescence quenching (t_f1/2_) and the recovery (t_r1/2_) half-times.

### Differential scanning calorimetry measurements

DSC was performed on isolated spinach thylakoid membranes suspended in buffer A, pH 7.5 or in buffer A, and pH 5.5 in the case of low-pH treated samples, which were washed twice in this buffer and additionaly incubated for 40 min prior to DSC scans. For recovery experiments the low-pH treated thylakoids were washed out twice and finally resuspended in buffer A, pH 7.5. Calorimetric measurements were performed on DASM-4 instrument, with 0.5 °C scanning rate and 1.3 mg/ml Chl content; for further details see^48^. Transition tempertures (denoted T_1_–T_4_) were determined at the peaks of the excess heat capacity curve and calorimetric enthalpy (ΔH_cal_) was estimated from the integrated area of the DSC curve.

### Determination of violaxanthin de-epoxidation by HPLC analysis

The concentration of the xanthophyll cycle pigments was determined by HPLC pigment analysis. The pigments were extracted from thylakoid membranes by 80% acetone and analysed using an Agilent 1,200 HPLC–DAD system (Agilent, USA) equipped with a LiChroCART RP-18 (250 × 4 mm, 5 μm) chromatographic column (Merck, Germany) applying the eluent system and gradient programme described by^72^. Dark-adapted thylakoid membranes of appropriate pH were taken after 10 min of the dark period (control samples), and illuminated thylakoids, after 10 min of illumination (770 µmol photons m^−2^ s^−1^; after measurement of light-induced NPQ). The extent of Vx de-epoxidation was determined as de-epoxidation state of xanthophyll cycle pigments DEPS = (Zx + Ax)/(Vx + Ax + Zx) according to^73^.

### Statistical analysis

All statistical analysis was performed using Origin software (OriginLab Corporation, Northhampton, USA). All numerical values are shown as the means ± standard deviations; n = 3–7, unless otherwise specified. The data sets were compared by one-way ANOVA followed by testing of means using Fisher’s multiple comparison. Significant differences were considered at *P* < 0.05.

## Supplementary information

Supplementary Information.
