# Supplementary material for: Modulation of non-bilayer lipid phases and the structure and functions of thylakoid membranes: effects on the water-soluble enzyme violaxanthin de-epoxidase
Source: Sci Rep. 2020 Jul 20;10:11959. doi: 10.1038/s41598-020-68854-x (PMC7371714; doi:10.1038/s41598-020-68854-x)
Supplement: Supplementary file 1 — Supplementary Information. [file 41598_2020_68854_MOESM1_ESM.pdf]

Supplementary Information

Modulation of non-bilayer lipid phases and the structure and functions of plant thylakoid membranes

Ondřej Dlouhý[1], Irena Kurasová[1,2], Václav Karlický[1,2], Uroš Javornik[3], Primož Šket[3,4], Nia Z. Petrova[5], Sashka B. Krumova[5], Janez Plavec[3,4,6], Bettina Ughy[1,7*], Vladimír Špunda[1,2*] and Győző Garab[1,7*]

[1]Faculty of Science, University of Ostrava, Ostrava, Czech Republic

[2]Global Change Research Institute, Czech Acad. Sci., Brno, Czech Republic

[3]Slovenian NMR Center, National Institute of Chemistry, Ljubljana, Slovenia

[4]EN-FIST Center of Excellence, Ljubljana, Slovenia

[5]Department of Biomacromolecules and Biomolecular Interactions, Institute of Biophysics and Biomedical Engineering, Bulgarian Academy of Sciences, Sofia, Bulgaria

[6]Faculty of Chemistry and Chemical Technology, Ljubljana, Slovenia

[7]Biological Research Centre, Szeged, Hungary

[*]Corresponding authors: garab.gyozo@brc.hu, vladimir.spunda@osu.cz, ughy.bettina@brc.hu

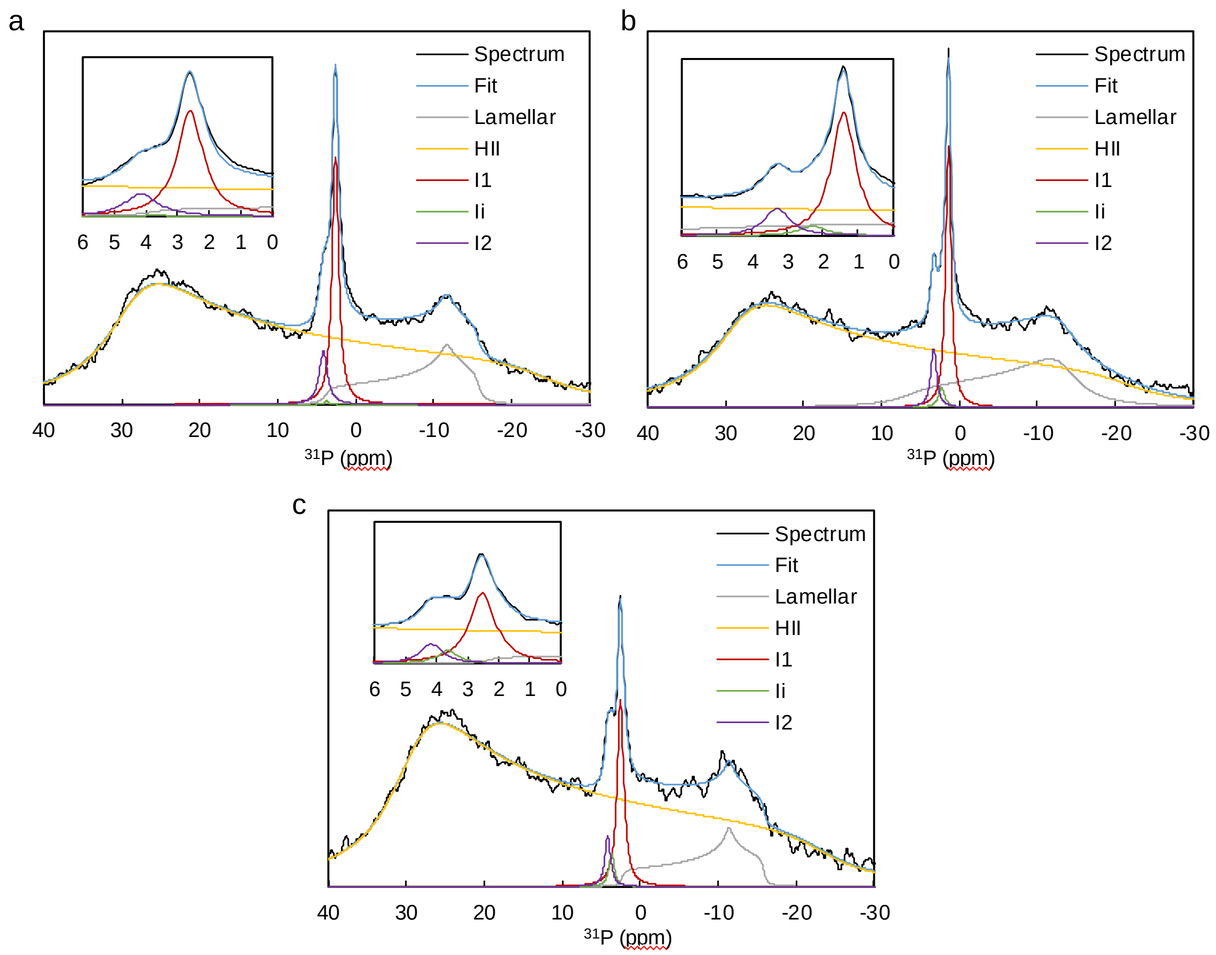

Supplementary Fig. 1: ^{31}P-NMR spectra measured on isolated spinach thylakoid membranes at different pH at 5 °C. (a) pH 7.5, **(b)** 5.5, and **(c)** 7.5 after a 5 min incubation time at pH 5.5 and then again at 7.5 (5.5-7.5). Sums of two measurements from independent experiments are shown, weigthed for Chl concentrations of **(a)** 9.7 mg/ml, **(b)** 11.1 mg/ml and **(c)** 8.3 mg/ml; the number of scans were 12 900 for each spectra. Insets, isotropic regions. The y-axis scale is identical for all spectra, including insets.

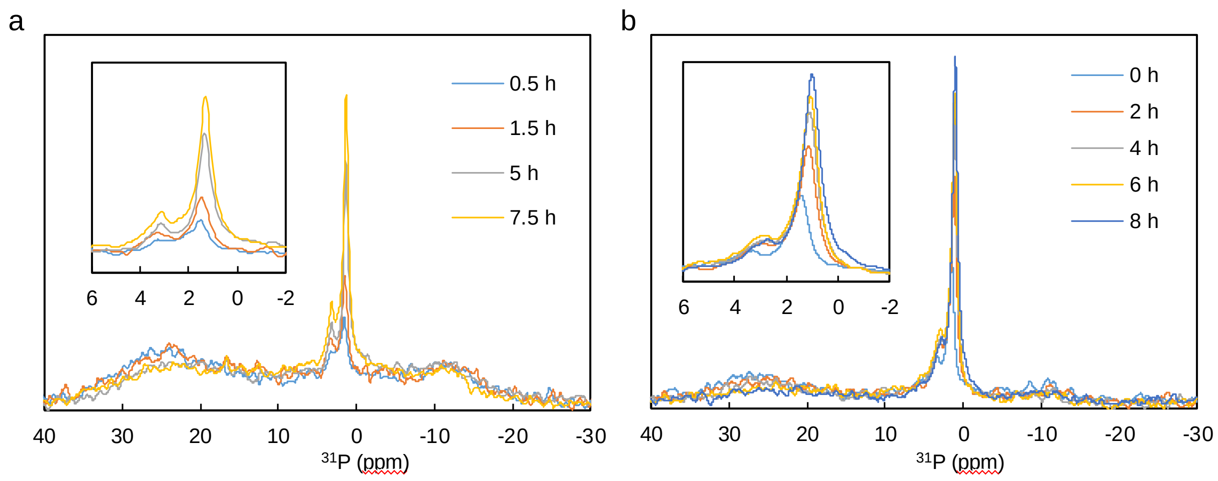

Supplementary Fig. 2: ^{31}P-NMR spectra of isolated spinach thylakoid membranes in different reaction media. Spectra obtained during storage of the membranes at 5 °C and pH 5.5 in **(a)** sorbitol- and **(b)** NaCl-based media. Chl concentrations were **(a)** 11.1 mg/ml, and **(b)** 13.6 mg/ml; both series of spectra were normalized to equal number of scans. The measurements were performed on the Avance Neo 600 MHz NMR spectrometer (Bruker) (a), and on the DD2 600 MHz NMR spectrometer (Agilent) (b). Insets show the isotropic regions.

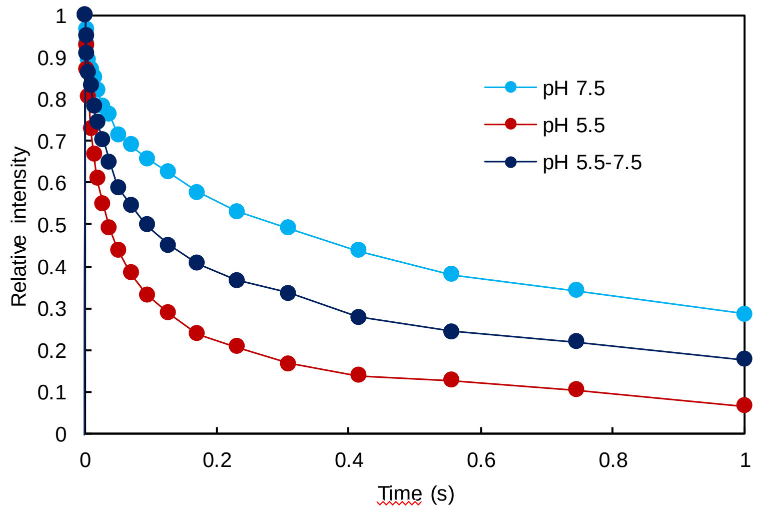

Supplementary Fig. 3: The effect of low-pH treatment on the permeability of isolated spinach thylakoid membranes, as reflected by electrochromic absorbance transients. Typical ΔA_{515} kinetic traces recorded at pH 7.5, 5.5. and 7.5 after the pH 5.5 treatment of the membranes; the initial amplitudes were normalized to 1; Chl concentration, 20 µg/ml.

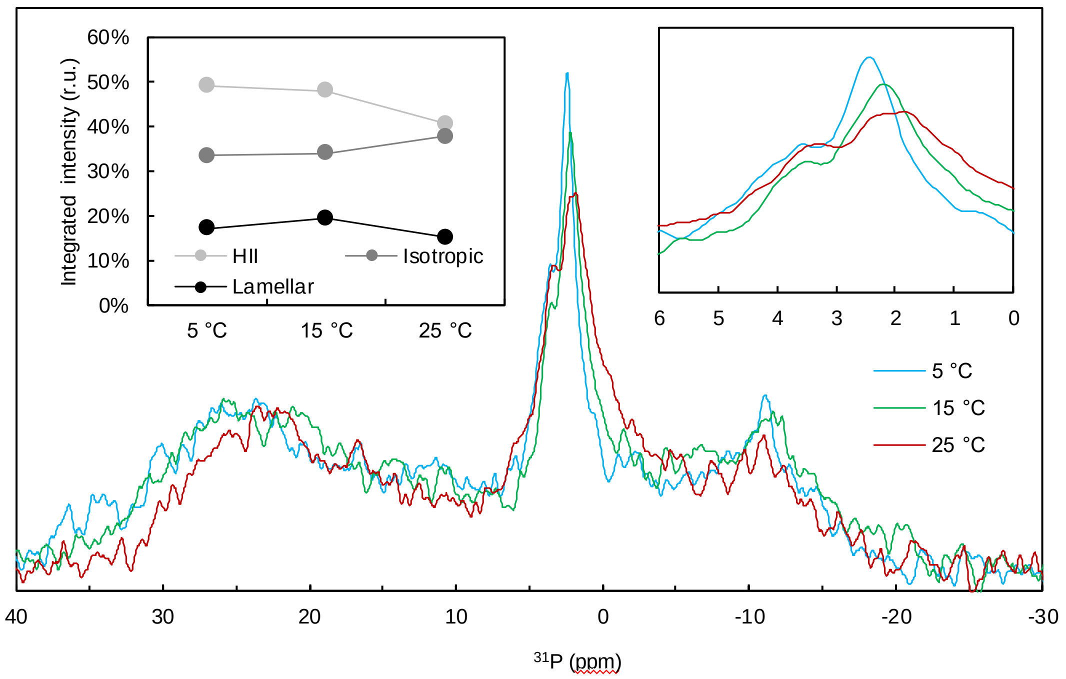

Supplementary Fig. 4: ^{31}P-NMR spectra of isolated spinach thylakoid membranes measured sequentially at 5, 15 and 25 °C. The Chl concentration was 11.4 mg/ml and the number of scans was 1 750 (ca 15 min) for each spectrum. Right inset, isotropic regions; left inset, integrated areas of different regions (H_{II} from 37 to 8 ppm; isotropic,from 7 to -7 ppm; lamellar from -7 to -20 ppm) relative to the total integralted areas

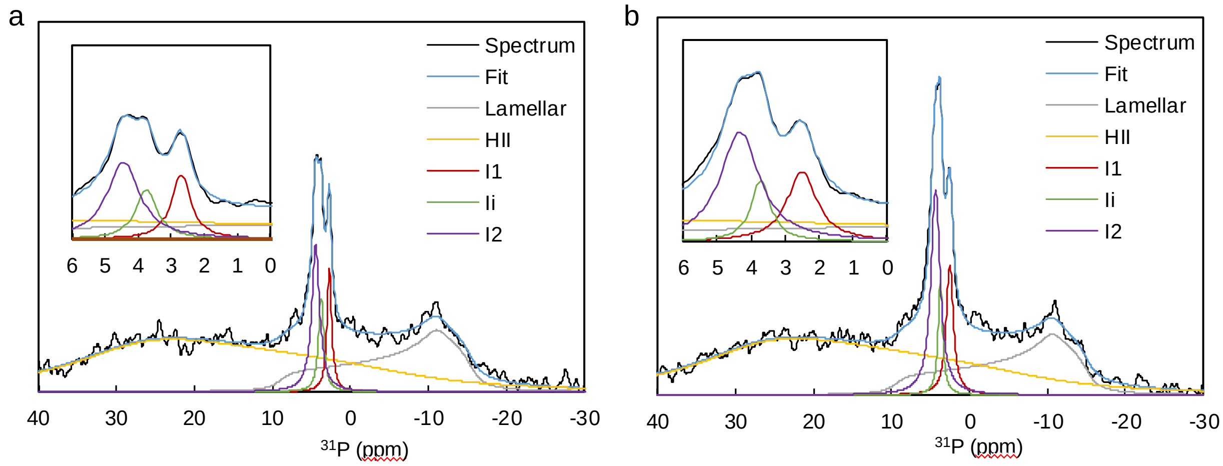

Supplementary Fig. 5: ^{31}P-NMR spectra of isolated spinach thylakoid membranes at (a) 5 and (b) 15 °C. The Chl concentration was 7.8 mg/ml and the number of scans was 1600 for both spectra. Also shown, the deconvoluted component spectra; insets, isotropic regions. The y-axis scale is identical for (a) and (b).

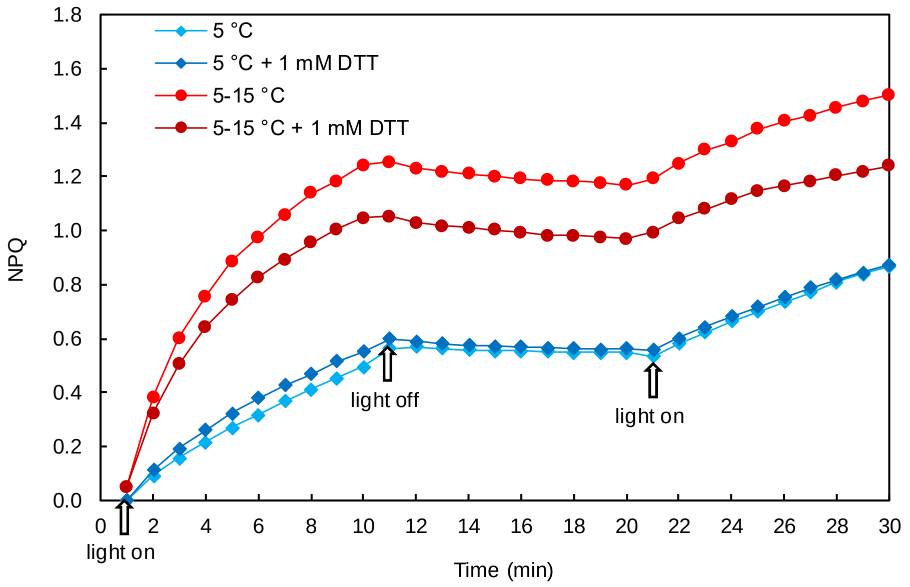

Supplementary Fig. 6: Typical NPQ induction and relaxation kinetics of isolated spinach thylakid membranes at 5 and 15 °C in the absence and presence of DTT. The membranes (25 µg/ml) were obtained from a series of experiments, during which the temperature of dense suspensions of isolated thylakoid membranes were cyclically switched between 5 and 15 °C; shown here, traces belonging to the first set; illumination (10 min), white light of 770 µmol photons m^{-2} s^{-1}; dark-relaxation, 10 min; DTT, when added, 1 mM.

Supplementary Table 1: Temperatures of denaturation of the well resolved sequential thermal transitions (denoted T1-T4) resolved in the DSC scans of thylakoid membranes measured at pH 7.5, 5.5 and recovered at 7.5, and calorimetric enthalpy (ΔH_{cal}), means ±SD.

pH	T1 (°C)	T2 (°C)	T3 (°C)	T4 (°C)	ΔH_{cal} (cal/g.deg)
**7.5**	56.8 (±1.0)	66.8 (±1.3)	75.4 (±1.9)	82.6 (±1.2)	27.6 (±6.1)
**5.5**	51.4 (±1.4)	64.6 (±1.3)		77.7 (±1.5)	27.9 (±6.0)
**5.5-7.5**	56.1 (±0.2)	68.1 (±0.1)	76.0 (±0.2)	83.3 (±0.1)	26.1 (±2.5)

Supplementary Table 2: Variations of the half-decay times ($t_{1/2}$) of the ΔA_{515} electrochromic absorbance changes and the relative amplitudes of the fast Chl-a fluorescence rise of isolated spinach thylakoid membranes during the cyclic variation of the temperature between 5 and 15 °C. Mean values and standard deviations from 3 independent experiments, normalized to the first measurements at 5 °C. $t_{1/2}$ at 5 °C was 1.01±0.32 s. The temperature treatments were performed on dense suspensions of the membranes; Chl concentration of the measurements, 20 µg/ml. Mean values and standard errors from of 3 independent experiments. Different letters in columns indicate statistically siginificant differences (ANOVA, F-test, P<0.05).

Temperature (°C)	Relative half-decay time (±SD)
5	100 (±0)^{a}
5-15	49 (±13)^{bc}
5-15-5	55 (±19)^{b}
5-15-5-15	24 (±8)^{cd}
5-15-5-15-5	28 (±11)^{bcd}
5-15-5-15-5-15	10 (±8)^{d}
